# Peripheral effects of morphine and expression of μ-opioid receptors in the dorsal root ganglia during neuropathic pain: nitric oxide signaling

**DOI:** 10.1186/1744-8069-7-25

**Published:** 2011-04-12

**Authors:** Arnau Hervera, Roger Negrete, Sergi Leánez, Jesús M Martín-Campos, Olga Pol

**Affiliations:** 1 Grup de Neurofarmacologia Molecular, Institut de Recerca de l'Hospital de la Sta Creu i Sant Pau & Institut de Neurociències, Universitat Autònoma de Barcelona, Barcelona, Spain; 2 Grup de Bioquímica, Institut de Recerca de l'Hospital de la Sta Creu i Sant Pau, Barcelona, Spain

## Abstract

**Background:**

The local administration of μ-opioid receptor (MOR) agonists attenuates neuropathic pain but the precise mechanism implicated in this effect is not completely elucidated. We investigated if nitric oxide synthesized by neuronal (NOS1) or inducible (NOS2) nitric oxide synthases could modulate the local antiallodynic effects of morphine through the peripheral nitric oxide-cGMP-protein kinase G (PKG)-ATP-sensitive K^+ ^(KATP) channels signaling pathway activation and affect the dorsal root ganglia MOR expression during neuropathic pain.

**Results:**

In wild type (WT) mice, the subplantar administration of morphine dose-dependently decreased the mechanical and thermal allodynia induced by the chronic constriction of the sciatic nerve (CCI), which effects were significantly diminished after their co-administration with different subanalgesic doses of a selective NOS1 (N-[(4S)-4-amino-5-[(2-aminoethyl)amino]pentyl]-N'-nitroguanidine tris(trifluoroacetate) salt; NANT), NOS2 (L-N(6)-(1-iminoethyl)-lysine; L-NIL), L-guanylate cyclase (1H-[1,2,4]oxadiazolo[4,3-a]quinoxalin-1-one; ODQ), PKG ((Rp)-8-(para-chlorophenylthio)guanosine-3',5'-cyclic monophosphorothioate; Rp-8-pCPT-cGMPs) inhibitor or a KATP channel blocker (glibenclamide). The evaluation of the expression of MOR in the dorsal root ganglia from sham-operated and sciatic nerve-injured WT, NOS1 knockout (KO) and NOS2-KO mice at 21 days after surgery demonstrated that, although the basal mRNA and protein levels of MOR were similar between WT and both NOS-KO animals, nerve injury only decreased their expression in WT mice.

**Conclusions:**

These results suggest that the peripheral nitric oxide-cGMP-PKG-KATP signaling pathway activation participates in the local antiallodynic effects of morphine after sciatic nerve injury and that nitric oxide, synthesized by NOS1 and NOS2, is implicated in the dorsal root ganglia down-regulation of MOR during neuropathic pain.

## Background

Neuropathic pain is a clinical manifestation characterized by the presence of allodynia and hyperalgesia and it is difficult to treat with the most potent analgesic compounds. Recent studies have demonstrated that the peripheral administration of μ-opioid receptor (MOR) agonists elicits antinociception in different models of neuropathic pain [1,2] and that their expression decreases after nerve injury [2,3]. Even so, the precise mechanisms implicated in the peripheral actions of morphine as well as in the expression of MOR during neuropathic pain are not completely elucidated.

Several studies have shown that nitric oxide, synthesized by neuronal (NOS1) or inducible (NOS2) nitric oxide synthases, mediates numerous neuropathic pain symptoms via central and peripheral nitric oxide-cGMP-PKG pathway activation [4-6]. Accordingly, the expression of NOS1 and NOS2 is up-regulated in the spinal cord and dorsal root ganglia of animals with neuropathic pain [7,8]. Moreover, the mechanical and thermal allodynia induced by nerve injury was reversed by the administration of selective NOS, guanylate cyclase o PKG inhibitors and attenuated or abolished in NOS1 and NOS2 knockout (KO) animals [4,6,8-10].

It is well known that the peripheral nitric oxide-cGMP-protein kinase G (PKG)-ATP-sensitive K^+ ^(KATP) channels signaling pathway activation plays a critical role in the local antinociceptive effects of morphine during inflammatory pain [11-13] but not in the peripheral antinociceptive effects of δ-opioid receptor (DOR) agonists during neuropathic pain [6]. In addition, several studies also show that nitric oxide regulates the expression of MOR and DOR under several pain conditions [6,14,15] but the exact role of nitric oxide in the peripheral antinociceptive actions of morphine and expression of MOR during neuropathic pain is not known.

Thus, to study if the nitric oxide-cGMP-PKG-KATP peripheral pathway activation, triggered by NOS1 and NOS2, could modulate the local effects of morphine in nerve-injured wild type (WT) mice, at 21 days after the chronic constriction of the sciatic nerve (CCI), we evaluated: 1) the mechanical and thermal antiallodynic effects of the subplantar administration of morphine; 2) the reversibility of these effects by their local co-administration with a selective MOR antagonist, D-Phe-Cys-Tyr-D-Trp-Arg-Thr-Pen-Thr-NH2 (CTAP) or a peripheral non-selective opioid receptor antagonist, naloxone methiodide (NX-ME); 3) the mechanical and thermal antiallodynic effects of a high dose of morphine co-administered with different subanalgesic doses of a selective NOS1 (N-[(4S)-4-amino-5-[(2-aminoethyl)amino]pentyl]-N'-nitroguanidine tris(trifluoroacetate) salt; NANT), NOS2 (L-N(6)-(1-iminoethyl)-lysine; L-NIL), soluble guanylate cyclase (1*H*-[1,2,4]oxadiazolo[4,3-*a*]quinoxalin-1-one; ODQ), PKG ((Rp)-8-(para-chlorophenylthio)guanosine-3',5'-cyclic monophosphorothioate; Rp-8-pCPT-cGMPs) inhibitor or a KATP channel blocker (glibenclamide).

To evaluate the role played by nitric oxide, synthesized by NOS1 and NOS2, in the peripheral expression of MOR during neuropathic pain, the mRNA and protein levels of MOR in the dorsal root ganglia of sciatic nerve-injured WT, NOS1-KO and NOS2-KO mice, at 21 days after surgery, were also assessed.

## Results

### Expression of neuropathic pain in WT mice

In accordance to our previous reports [6,8], the total sciatic nerve ligation produced unilateral mechanical allodynia and thermal allodynia at 21 days after surgery. Thus, sciatic nerve injury led to a significant decrease in the percentage of the basal response of the threshold for evoking paw withdrawal to a mechanical stimulus in the ipsilateral paw of sciatic nerve-injured animals (37.4 ± 3.5) as compared to their contralateral paw (100.0 ± 6.3) as well as to the contralateral (104.5 ± 4.7) and ipsilateral (93.5 ± 9.1) paws of sham-operated mice (*P *< 0.001; one-way ANOVA followed by the Student Newman Keuls test). Similar results has been obtained for thermal allodynia where a significant increase in the number of paw elevations to cold thermal stimulus in the ipsilateral paw of sciatic nerve-injured animals (5.7 ± 0.6) as compared to their contralateral paw (0.2 ± 0.2) as well as to the contralateral (0.3 ± 0.2) and ipsilateral (0.2 ± 0.2) paws of sham-operated mice, has been also demonstrated (*P *< 0.001; one-way ANOVA followed by the Student Newman Keuls test).

### Effects of the subplantar administration of morphine in the mechanical and thermal allodynia induced by sciatic nerve injury in WT mice and reversal of their effects by CTAP or NX-ME

**Figure 1:**
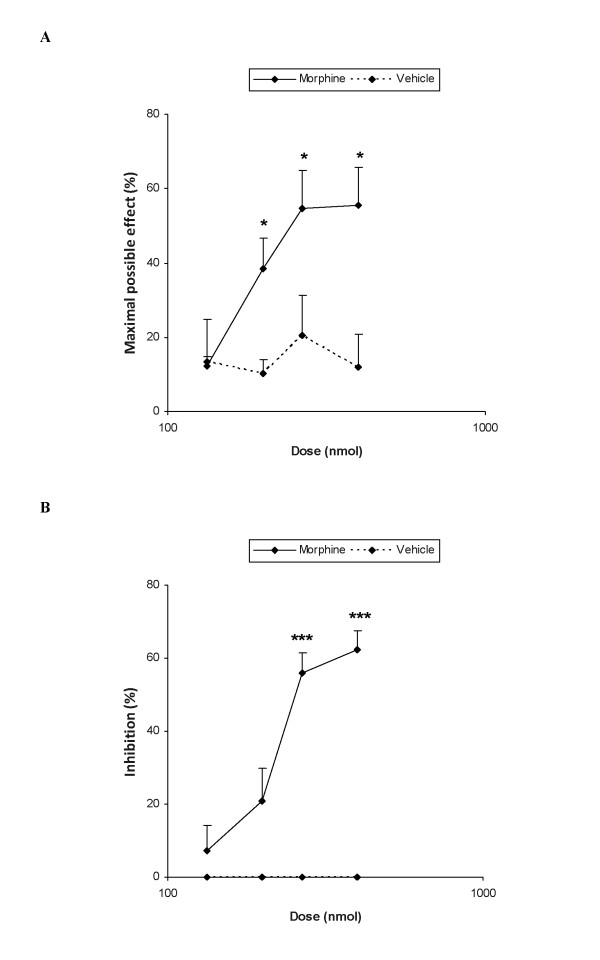
**Antiallodynic effects of morphine**. Effects of the subplantar administration of different doses (logarithmic axis) of morphine or vehicle on the mechanical (A) and thermal allodynia (B) induced by CCI in the ipsilateral paw of WT mice at 21 days after surgery. Morphine was administered 20 min before starting behavioral testing. Data are expressed as mean values of maximal possible effect (%) for mechanical allodynia and inhibition (%) for thermal allodynia ± SEM (5-6 animals for dose). In both tests, for each dose, * *P <*0.05 and *** *P <*0.001 denote significant differences between morphine and vehicle treated animals (Student's t test).

The subplantar administration of morphine into the ipsilateral paw dose-dependently inhibited the mechanical (Figure 1A) and thermal (Figure 1B) allodynia induced by the chronic constriction of the sciatic nerve. Thus, the mechanical and thermal antiallodynic effects produced by high doses of morphine in the ipsilateral paw of sciatic nerve-injured WT mice were significantly higher than those obtained in their corresponding vehicle treated groups (*P <*0.05; Student's t test). Moreover, analyzing the ED_50 _values our data showed that the potency of morphine on the inhibition of mechanical, 194.9 nmol (148.7-255.9) and thermal sensitivity, 225.9 nmol (191.0-267.1) induced by sciatic nerve injury was very analogous.

The subplantar administration of morphine or vehicle did not elicit any significant antinociceptive effect neither in the contralateral paw of sciatic nerve-injured mice nor in the ipsilateral or contralateral paw of sham-operated mice (data not shown).

**Figure 2:**
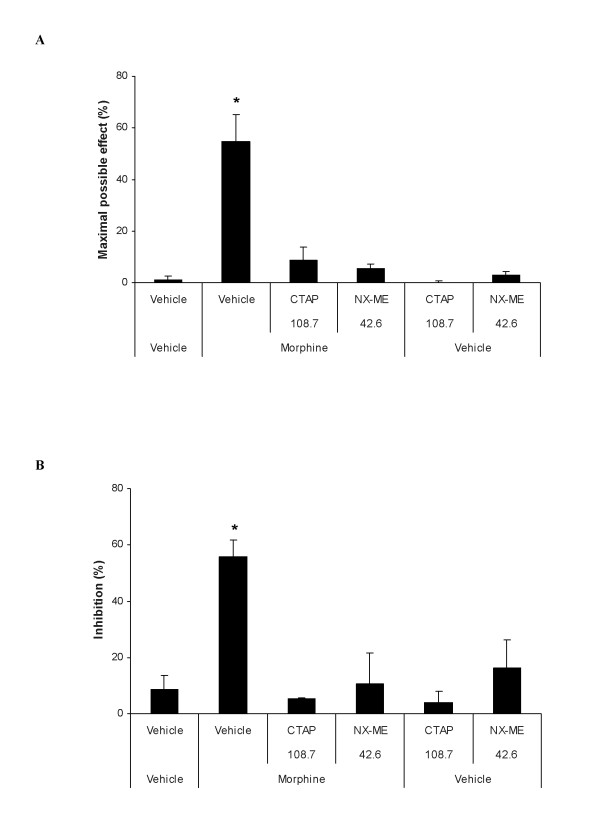
**Reversion of the antiallodynic effects of morphine**. Reversal of the effects of morphine (400 nmol) on the mechanical (A) and thermal (B) allodynia induced by CCI in the ipsilateral paw of WT mice, at 21 days after CCI, by the subplantar co-administration of a selective MOR antagonist (CTAP; 108.7 nmol) or a peripheral non-selective opioid receptor antagonist (NX-ME; 42.6 nmol). The effects of the subplantar administration of vehicle, CTAP (108.7 nmol) or NX-ME (42.6 nmol) administered alone are also shown. Data are expressed as mean values of maximal possible effect (%) for mechanical allodynia and inhibition (%) for thermal allodynia ± SEM (5-6 animals for each group). For each test, * represents significant differences compared to the other groups (*P <*0.05; one way ANOVA, followed by the Student Newman Keuls test).

The mechanical (Figure 2A) and thermal (Figure 2B) antiallodynic effects produced by morphine (400 nmol) in the ipsilateral paw of sciatic nerve-injured WT mice were completely reversed by the subplantar co-administration with a selective MOR (CTAP, 108.7 nmol) or the non-selective peripherally acting opioid receptor (NX-ME, 42.6 nmol) antagonist (*P <*0.001; one way ANOVA followed by the Student Newman Keuls test). The subplantar administration of vehicle, CTAP or NX-ME alone in sciatic nerve-injured and sham-operated WT mice did not show any significant effect on the two different nociceptive responses evaluated in this study.

### Involvement of the peripheral nitric oxide-cGMP-PKG-KATP signaling pathway triggered by NOS1 and NOS2 in local antiallodynic effects produced by morphine after the sciatic nerve injury in WT mice

The role of the peripheral nitric oxide-cGMP-PKG-KATP signaling pathway, activated by NOS1 and NOS2, in the local mechanical and thermal antiallodynic effects produced by morphine during neuropathic pain was assessed by evaluating the effects produced by a high dose of morphine (400 nmol) co-administered with different dose of NANT, L-NIL, ODQ, Rp-8-pCPT-cGMPs, glibenclamide or vehicle in sciatic nerve-injured WT mice at 21 days after surgery.

**Figure 3:**
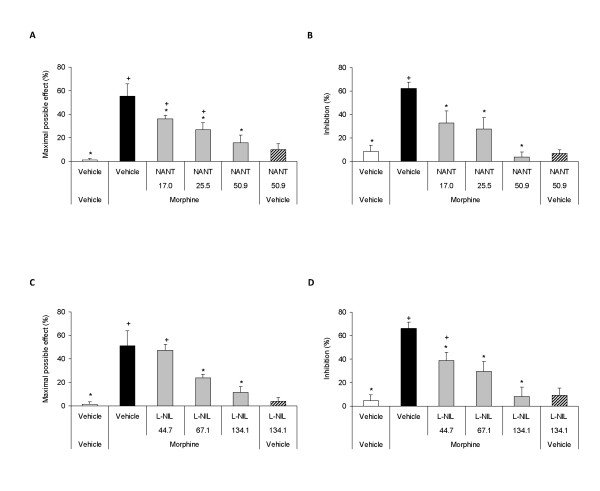
**Role of the peripheral nitric oxide synthesized by NOS1 and NOS2 in the antiallodynic effects of morphine**. Mechanical (A, C) and thermal (B, D) antiallodynic effects of the subplantar co-administration of morphine (400 nmol) plus vehicle or different doses of NANT (17.0 - 50.9 nmol; A, B) or L-NIL (44.7 - 134.1 nmol; C, D) in the ipsilateral paw of sciatic nerve-injured WT mice at 21 days after surgery. The effects of the subplantar administration of vehicle and the maximal doses of NANT (50.9 nmol) or L-NIL (134.1 nmol) injected alone are also shown. All drugs were administered 20 min before starting behavioral testing. Data are expressed as mean values of the maximal possible effect (%) for mechanical allodynia and as inhibition (%) for thermal allodynia ± SEM (5-6 animals per group). For each behavioral test and selective inhibitor assayed, * *P <*0.05 denotes significant differences vs. group treated with morphine plus vehicle (one way ANOVA followed by Student Newman Keuls test) and + *P <*0.05 denotes significant differences vs. group treated with vehicle (one way ANOVA followed by the Student Newman Keuls test).

**Figure 4:**
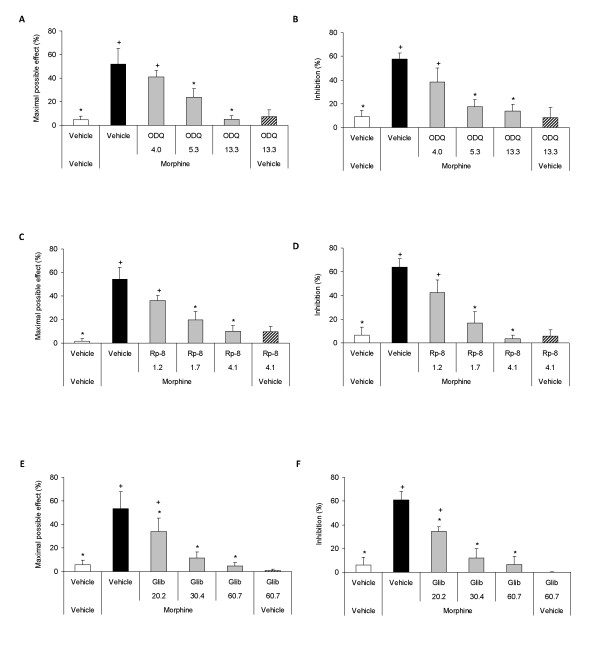
**Role of the peripheral nitric oxide-cGMP-PKG-KATP signaling pathway in the antiallodynic effects of morphine**. Mechanical (A, C, E) and thermal (B, D, F) antiallodynic effects of the subplantar co-administration of morphine (400 nmol) plus vehicle or different doses of ODQ (4.0 - 13.3 nmol; A, B), Rp-8 (1.2 - 4.1 nmol; C, D) or glibenclamide (Glib; 20.2 - 60.7 nmol; E,F) in the ipsilateral paw of sciatic nerve-injured WT mice at 21 days after surgery. The effects of the subplantar administration of vehicle and the maximal doses of ODQ (13.3 nmol), Rp-8 (4.1 nmol) or glibenclamide (60.7 nmol) injected alone are also shown. All drugs were administered 20 min before starting behavioral testing. Data are expressed as mean values of the maximal possible effect (%) for mechanical allodynia and as inhibition (%) for thermal allodynia ± SEM (5-6 animals per group). For each behavioral test and selective inhibitor assayed, * *P <*0.05 denotes significant differences vs. group treated with morphine plus vehicle (one way ANOVA followed by the Student Newman Keuls test) and + *P <*0.05 denotes significant differences vs. group treated with vehicle (one way ANOVA followed by Student Newman Keuls test).

Our results showed that the local mechanical and thermal antiallodynic effects of morphine in the ipsilateral paw of sciatic nerve-injured WT mice were inhibited by their peripheral co-administration with NANT or L-NIL (Figure 3) as well as with ODQ, Rp-8-pCPT-cGMPs or glibenclamide (Figure 4) in a dose-dependent manner (*P <*0.001, one way ANOVA followed by Student Newman Keuls test). Moreover, the local co-administration of morphine plus NANT, L-NIL, ODQ, Rp-8-pCPT-cGMPs or glibenclamide did not have any significant effect neither on the contralateral paw of sciatic nerve-injured mice nor in the ipsilateral or contralateral paw of sham-operated animals (data not shown).

Our results also indicated that the subplantar administration of the highest doses of NANT (50.9 nmol) or L-NIL (134.1 nmol; Figure 3) as well as of ODQ (13.3 nmol), Rp-8-pCPT-cGMP (4.1 nmol) or glibenclamide (60.7 nmol; Figure 4) administered alone did not produce any significant antiallodynic effect on the ipsilateral paws of sciatic nerve-injured WT mice as compared to vehicle group. Moreover, the subplantar administration of these doses of NANT, L-NIL, ODQ, Rp-8-pCPT-cGMPs or glibenclamide as well as of vehicle did not have any significant antinociceptive effect neither on the contralateral paw of sciatic nerve-injured mice nor in the ipsilateral or contralateral paw of sham-operated animals (data not shown).

### The expression of MOR in the dorsal root ganglia of sciatic nerve-injured WT, NOS1-KO and NOS2-KO mice

**Figure 5:**
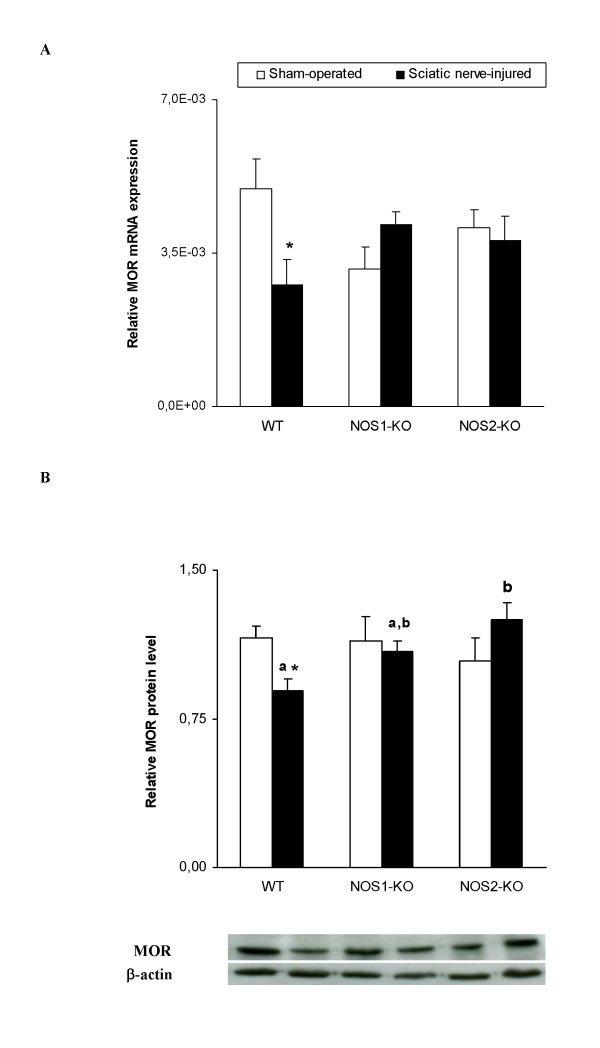
**Dorsal root ganglia expression of MOR in WT, NOS1-KO and NOS2-KO mice**. Relative mRNA (A) and protein (B) expression of MOR in the ipsilateral dorsal root ganglia from sham-operated or sciatic nerve-injured WT, NOS1-KO and NOS2-KO mice. Data are expressed as mean values ± SEM (4-5 samples per group). For each genotype, * *P <*0.05 denotes significant differences between sciatic nerve-injured and sham-operated mice (Student's t test). For each experimental group, different letters (a, b) denote significant differences between genotypes (*P <*0.05; one way ANOVA followed by the Student Newman Keuls test).

The mRNA and protein levels of MOR in the dorsal root ganglia of WT and both NOS-KO mice are shown in Figure 5A and 5B, respectively. Although the two way ANOVA did not show any effect of the genotype or surgery, a significant interaction between theme was demonstrated for mRNA (*P <*0.037) and protein (*P <*0.029) expression. Thus, while sciatic nerve injury significantly decreases the MOR mRNA (*P <*0.043, Student's t test) and protein (Student's t test, *P <*0.002) levels in WT mice, it did not change their expression in both KO mice when comparing sciatic nerve-injured vs. sham-operated animals.

In addition, non significant differences were found between genotypes when compared the expression of MOR mRNA among theme in sham-operated or sciatic nerve-injured mice, although a significant increase in the MOR protein levels were observed in sciatic nerve-injured mice when compared NOS2-KO vs. WT animals (one way ANOVA, *P <*0.014).

## Discussion

The present results demonstrate for first time that the local administration of morphine dose-dependently inhibited the mechanical and thermal allodynia induced by sciatic nerve injury through the activation of the peripheral nitric oxide-cGMP-PKG-KATP signaling pathway. This study also shows that nitric oxide, synthesized by NOS1 and NOS2, is implicated in the peripheral down-regulation of MOR during neuropathic pain.

In a model of CCI-induced neuropathic pain, our results confirmed the mechanical antiallodynic effects of MOR agonists locally administered [1,2] and further demonstrated the thermal antiallodynic effects produced by morphine in these experimental conditions with a similar effectiveness. The specificity and the peripheral antiallodynic effects of morphine after sciatic nerve injury was demonstrated by the complete reversion of their effects with a selective MOR antagonist (CTAP) and a non-selective peripherally acting opioid receptor antagonist (NX-ME), which did not have any effect when were administered alone. In addition, the highest dose of morphine did not produce any significant effect in the contralateral paw of sciatic nerve-injured mice, supporting the peripheral site of action of this drug.

A clear relationship between the antinociceptive effects of MOR agonists and the peripheral nitric oxide-cGMP-PKG-KATP signaling pathway activation during inflammatory pain has been extensively demonstrated [11-13,16]. Our results show for first time, that the local administration of morphine alleviates the mechanical and thermal allodynia induced by sciatic nerve injury through the activation of the peripheral nitric oxide-cGMP-PKG signaling pathway, triggered by NOS1 and NOS2, which culminate in an increased activation of KATP channels causing the hyperpolarization of nociceptive neurons. Indeed, the local mechanical and thermal antiallodynic effects produced by a high dose of morphine in sciatic nerve-injured WT mice were dose-dependently diminished with their co-administration with subanalgesic doses of L-NIL, NANT, ODQ, Rp-8-pCPT-cGMPs or glibenclamide. These results are in contrast to the reduced nociceptive responses induced by morphine after their intrathecal co-administration with several nitric oxide-cGMP-PKG-MAPKs inhibitors [17]. These findings support the idea that the nitric oxide-cGMP pathway may play different roles depending on the site of action, whereas peripheral activation produces antinociception, their central activation should be causing of nociceptive behaviors [18]. In addition, while the peripheral administration of morphine causes antinociception by the activation of the peripheral nitric oxide-cGMP signaling pathway which culminate in an increased activation of KATP channels causing the hyperpolarization of nociceptive neurons [13], their intrathecal administration produces nociception by the activation of the spinal nitric oxide-cGMP signaling pathway that culminate in an increased activation of MAPKs which increases membrane excitability and induces spinal neuronal sensitization [19]. Moreover, the results of the present study are also in contrast to the enhanced antinociceptive effects of a DOR agonist after their co-administration with peripheral nitric oxide synthases or cGMP-PKG pathway blockers in sciatic nerve-injured animals [6]. Therefore, our findings demonstrate that while MOR agonists use the same mechanism of action to produce peripheral antinociception during inflammatory and neuropathic pain with different effectiveness, DOR agonists did not active the same way to produce peripheral antinociception in both types of pain, although a comparable potency was maintained [2,6]. Thus, a possible explanation for the reduced effectiveness of locally administered MOR agonists during neuropathic pain as compared to inflammatory, apart from the different alterations in the expression of MOR that occurs after peripheral inflammation (increases) or nerve injury (decreases) [2], might be also related to the drastic reduction in the peripheral KATP channels described in nerve-injured animals [20].

Several studies have demonstrated the involvement of nitric oxide in the regulation of opioid receptor gene transcription after peripheral inflammation and nerve injury [6,21,22]. In this report, we have investigated the role played by nitric oxide, synthesized by NOS1 and NOS2, in the decreased expression of MOR after neuropathic pain by using knockout mice for these enzymes. Our results showed that, although the basal dorsal root ganglia mRNA and protein levels of MOR were similar between WT and NOS-KO animals, nerve injury only decreased the MOR expression in WT mice. These findings suggest that nitric oxide, derived from NOS1 and NOS2, is implicated in the peripheral down-regulation of MOR after sciatic nerve-injury. Therefore and according to what occurs with the peripheral actions of morphine during inflammatory and neuropathic pain, these molecular data also support the evidence of the dual role played by nitric oxide in the modulation of the expression of MOR in both pain models. That is, while nitric oxide increases the peripheral expression of MOR during inflammation, it decreases their expression after nerve injury.

In summary, our data demonstrate that the activation of the nitric oxide-cGMP-PKG-KATP signaling peripheral pathway participates in the local antiallodynic effects produced by morphine during sciatic nerve injury and that nitric oxide, synthesized by NOS1 and NOS2, is involved in the decreased expression of MOR during neuropathic pain.

## Conclusions

The present study demonstrates for first time that morphine can effectively attenuate neuropathic pain through the activation of the peripheral nitric oxide-cGMP-PKG-KATP signaling pathway and the decreased expression of MOR after sciatic nerve injury is regulated by nitric oxide. These data contribute to a better comprehension of the mechanism through peripheral MOR agonists produce antinociception after nerve injury and provide new insights into the development of novel therapeutic approaches for alleviating neuropathic pain.

## Methods

### Animals

Male NOS1-KO (C57BL/6J background) and NOS2-KO mice (C57BL/6J background) were purchased from Jackson Laboratories (Bar Harbor, ME, USA) while WT mice with the same genetic background (C57BL/6J) were acquired from Harlan Laboratories (Barcelona, Spain). All mice weighing 21 to 25 g were housed under 12-h/12-h light/dark conditions in a room with controlled temperature (22°C) and humidity (66%). Animals had free access to food and water and were used after a minimum of 6 days acclimatization to the housing conditions.

Animal procedures were conducted in accordance with the guidelines of the UK Animals Act 1986 (Scientific Procedures) and the guidelines of the European Communities Directive 86/609/EEC regulating animal research. The study protocol was approved by the local Committee of Animal Use and Care of the Autonomous University of Barcelona. All experiments were performed under blind conditions.

### Induction of neuropathic pain

Neuropathic pain was induced by the chronic constriction of the sciatic nerve [6,8]. Briefly, sciatic nerve ligation was performed under isoflurane anesthesia (3% induction, 2% maintenance). The biceps femoris and the gluteus superficialis were separated by blunt dissection, and the right sciatic nerve was exposed. The injury was produced by tying three ligatures around the sciatic nerve as described by Bennett and Xie [23]. The ligatures (4/0 silk) were tied loosely around the nerve with 1 mm spacing, until they elicited a brief twitch in the respective hindlimb, which prevented over-tightening of the ligations, taking care to preserve epineural circulation. Sham-operated mice that underwent exposure of the right sciatic nerve without ligature were used as controls.

The development of mechanical and thermal allodynia was evaluated by using the von Frey filaments and cold plate tests, respectively. All animals were tested in each paradigm before surgery and at 21 days after CCI.

### Nociceptive behavioral tests

**Mechanical allodynia **was quantified by measuring the hind paw withdrawal response to von Frey filament stimulation. In brief, animals were placed in a Plexiglas^® ^box (20 cm high, 9 cm diameter) with a wire grid bottom through which the von Frey filaments (North Coast Medical, Inc., San Jose, CA, USA) bending force range from 0.008 to 3.5 g, were applied by using a modified version of the up-down paradigm, as previously reported by Chaplan [24]. The filament of 0.4 g was used first and the 3.5 g filament was used as a cut-off. Then, the strength of the next filament was decreased or increased according to the response. The threshold of response was calculated from the sequence of filament strength used during the up-down procedure by using an Excel program (Microsoft Iberia SRL, Barcelona, Spain) that includes curve fitting of the data. Clear paw withdrawal, shaking or licking of the paw were considered nociceptive-like responses. Both ipsilateral and contralateral hind paws were tested. Animals were allowed to habituate for 1 h before drug administration in order to allow an appropriate behavioral immobility.

**Thermal allodynia **to cold stimulus was assessed by using the hot/cold-plate analgesia meter (Ugo Basile, Italy), previously described by Bennett and Xie [23]. The number of elevations of each hind paw was recorded in the mice exposed to the cold plate (4 ± 0.5°C) for 5 minutes.

### Molecular experiments

#### Tissue isolation

Sham-operated and sciatic nerve-injured WT, NOS1-KO and NOS2-KO mice were sacrificed at 21 days after surgery by cervical dislocation. Three dorsal root ganglia from the ipsilateral lumbar section (L3 to L5) were collected from each animal. They were removed immediately after sacrifice, frozen in liquid nitrogen and stored at -80°C until assay. Because of the small size of the unilateral dorsal root ganglia, tissues from three to five animals were pooled together to obtain enough RNA or protein levels for performing the real time-PCR or Western blot analysis, respectively.

#### Total RNA extraction and reverse transcription

Tissues were homogenized in ice-cold with a homogenizer (Ultra-Turf, T8; Ika Werke, Staufen, Germany) and the total RNA was extracted with TRIzol reagent (Invitrogen, Renfrewshire, England). The amount of the purified RNA (A_260_/A_280 _ratio ≥ 1.9) was determined by spectrophotometry. In all experiments, 1 μg of total RNA was reverse transcribed into cDNA using SuperScript II RNAse H^- ^reverse transcriptase (Invitrogen, Renfrewshire, UK) in a final volume of 10 μl. Negative controls were performed in which all of the components were included except reverse transcriptase.

#### TaqMan probe real-time polymerase chain reaction (PCR)

The expression of MOR mRNA was determined by real-time PCR using a designed mice TaqMan^® ^gene expression assay (Applied Biosystems, CA, USA) for this gene (MU-OR1-E2E3). A probe against GAPDH (Mm 99999915_g1) was used as endogenous control and reactions without RNA were included as negative controls to ensure the specificity. PCR reactions were set up in 96-well plates containing the corresponding cDNA, 0.9 μmol/L of each forward and reverse primers, 0.25 μmol/L of TaqMan^® ^MGB probe and a final concentration of 1× universal master mix (Applied Biosystems, CA, USA), which provides the PCR buffer, MgCl_2_, dNTPs, and the thermal stable AmpliTaq Gold DNA polymerase. The assay was conducted using the Applied Biosystems ABI PRISM 7000 Sequence Detection System. All samples were assayed in duplicate. Relative expression of the target gene was calculated by means of the comparative threshold cycle (CT) method [25].

#### Western blot analysis

The MOR protein levels were analyzed by Western blot. Tissues were homogenized in ice-cold lysis buffer (50 mM Tris·Base, 150 nM NaCl, 1% NP-40, 2 mM EDTA, 1 mM phenylmethylsulfonyl fluoride, 0.5 Triton X-100, 0.1% SDS, 1 mM Na_3_VO_4_, 25 mM NaF, 0.5% protease inhibitor cocktail, 1% phosphatase inhibitor cocktail). All reagents were purchased at Sigma (St. Louis, MO, USA) with the exception of NP-40 from Calbiochem. The crude homogenate was solubilised 1 hour at 4°C, sonicated for 10 seconds and centrifugated at 4°C for 15 min at 700 × g. The supernatants (80 μg of total protein) were mixed with 4 × laemmli loading buffer and then loaded onto 4% stacking/10% separating SDS-polyacrylamide gels. The proteins were electrophoretically transferred onto PVDF membrane for 90 minutes, blocked with PBST + 5% nonfat dry milk, and subsequently incubated overnight at 4°C with a polyclonal rabbit anti-MOR antibody (1:1.000, Chemicon, Millipore). The proteins were detected by a horseradish peroxidase-conjugated anti-rabbit secondary antibody (GE Healthcare, Little Chalfont, Buckinghamshire, UK) and visualized by chemiluminescence reagents provided with the ECL kit (Amersham Pharmacia Biotech, Piscataway, NJ, USA) and exposure onto hyperfilm (GE, Healthcare). The intensity of blots was quantified by densitometry. The membrane was stripped and reapplied with a monoclonal rabbit anti-β-actin antibody (1:10.000, Sigma, St. Louis, MO, USA) used as a loading control.

### Experimental protocol

In a first set of experiments we assessed the expression of neuropathic pain by using the mouse model of CCI previously used by us [6,8]. After the habituation period, baseline responses were established in the following sequence: von Frey filaments and cold plate tests. After that, neuropathic pain was induced and animals were again tested in each paradigm at 21 days after surgery before and after drug administration. Sham-operated mice were used as controls.

Due to the lack of mechanical and thermal allodynia induced by CCI in NOS1-KO and NOS2-KO mice, as we have previously demonstrated [8], the mechanical and thermal antiallodynic effects produced by the subplantar administration of different doses of morphine (133-400 nmol) or saline in the ipsilateral and contralateral paws of sciatic nerve-injured and sham-operated animals, were only evaluated in WT mice.

In another set of experiments, the specificity of the mechanical and thermal antiallodynic effects produced by a high dose of morphine (400 nmol) in sciatic nerve-injured WT mice at 21 days after surgery, was assessed by evaluating the reversibility of their effects with the peripheral co-administration with a selective MOR antagonist (CTAP; 108.7 nmol) or a peripheral non-selective opioid receptor antagonist (NX-ME; 42.6 nmol). The effects of these antagonists administered alone were also tested in sciatic nerve-injured and sham-operated WT mice at 21 days after surgery.

The possible involvement of the peripheral nitric oxide-cGMP-PKG- KATP signaling pathway, activated by NOS1 and NOS2, in the local mechanical and thermal antiallodynic effects of a MOR agonist has been evaluated in an extra group of WT mice. For this purpose, the local effects produced by different doses of NANT (17.0-50.9 nmol) a selective NOS1 inhibitor [26], L-NIL (44.7-134.1 nmol) a selective NOS2 inhibitor [27], ODQ (4.0-13.3 nmol) a selective soluble guanylyl cyclase inhibitor [28], Rp-8-pCPT-cGMPs (1.2-4.1 nmol) a PKG inhibitor [29], glibenclamide (20.2-60.7 nmol) a KATP channel blocker [30] or vehicle administered alone or combined with a high dose of morphine (400 nmol) in the ipsilateral and contralateral paws of sciatic nerve-injured and sham-operated WT mice at 21 days after surgery, were also evaluated. The doses of all tested inhibitors were selected according to our previous experiments as the ones which did not produce a significant antiallodynic effect after CCI-induced neuropathic pain [6].

In all experiments, antinociception in Von Frey filaments is expressed as the percentage of maximal possible effect, where the test latencies pre (baseline) and post drug administration are compared and calculated according to the following equation:

In the cold plate test, the inhibitory effects were calculated according to the following equation:

Finally, the mRNA and protein levels of MOR in the ipsilateral site of the dorsal root ganglia from sciatic nerve-injured or sham-operated WT, NOS1-KO and NOS2-KO mice at 21 after surgery were also evaluated by using real time PCR and western blot, respectively.

### Drugs

Morphine-HCl was obtained from Alcaiber S.A. (Madrid, Spain) and L-NIL from Tocris (Ellisville, MI). CTAP, NX-ME, NANT, ODQ, Rp-8-pCPT-cGMPs and glibenclamide were purchase from Sigma-Aldrich (St. Louis, MO). Morphine, CTAP, NX-ME, NANT, L-NIL and Rp-8-pCPT-cGMPs were dissolved in saline solution (0.9% NaCl) while ODQ and glibenclamide in dimethyl sulfoxide (DMSO; 10% and 50% solution in saline, respectively). All drug combinations were diluted in the highest required concentration of DMSO. All drugs alone or combined were injected in a final volume of 30 μl. In all experiments, drugs were administered into the plantar side of the right paw, 20 min before behavioral testing. For each group treated with a drug the respective control group received the same volume of vehicle.

### Statistical analysis

Data are expressed as mean ± standard error of the mean (SEM). For each test and dose, the comparison of the effects produced by morphine vs. the effects produced by vehicle in the contralateral and ipsilateral paw of nerve-injured or sham-operated mice was evaluated by using a Student's t test. The ED_50 _values (dose that produced a 50% of the maximal effect) plus 95% confidence limits were determined by linear regression analysis of dose-response relations based on at least 5-6 mice per dose.

For each test, the reversion of the mechanical and thermal antiallodynic effects produced by morphine with CTAP or NX-ME and the effects produced by these antagonists administered alone in the ipsilateral paw of sciatic nerve-injured and sham operated WT mice were analyzed by using a one way ANOVA followed by the Student Newman Keuls test.

The comparison between the mechanical and thermal antiallodynic effects produced by a high dose of morphine subplantarly administered alone or combined with different doses of specific inhibitors (NANT, L-NIL, ODQ, Rp-8-pCPT-cGMPs or glibenclamide) in the ipsilateral paw of sciatic nerve-injured and sham-operated WT mice was performed by using a one way ANOVA followed by the Student Newman Keuls test.

Changes in the expression of MOR (mRNA or protein) in the dorsal root ganglia of sciatic nerve-injured and sham-operated WT, NOS1-KO and NOS2-KO mice at 21 after surgery, were analyzed by using a two-way ANOVA (genotype and surgery as between factors of variation), followed by the corresponding one way ANOVA or Student's t test when required. A value of *P <*0.05 was considered as a significant.

## Abbreviations

CCI: chronic constriction of the sciatic nerve; cGMP: guanosine 3',5'-cyclic monophosphate; CTAP: D-Phe-Cys-Tyr-D-Trp-Arg-Thr-Pen-Thr-NH2; DMSO: dimethyl sulfoxide; DOR: δ-opioid receptor; KATP: ATP-sensitive K^+ ^channels; KO: knockout; L-NIL: L-N(6)-(1-iminoethyl)-lysine; MOR: μ-opioid receptor; NANT: N-[(4S)-4-amino-5-[(2-aminoethyl)amino]pentyl]-N'-nitroguanidine tris (trifluoroacetate) salt; NOS1: neuronal nitric oxide synthase; NOS2: inducible nitric oxide synthase; NX-ME: naloxone methiodide; ODQ: 1*H*-[1,2,4]oxadiazolo[4,3-*a*]quinoxalin-1-one; PKG: cGMP-dependent protein kinase; Rp-8-pCPT-cGMPs: (Rp)-8-(para-chlorophenylthio)guanosine-3',5'-cyclic monophosphorothioate; SEM: standard error of the mean; WT: wild type.

## Competing interests

The authors declare that they have no competing interests.

## Authors' contributions

AH and OP conceived and designed the experiments. AH RN SL and JMMC performed the experiments. AH RN SL and JMMC analyzed the data. AH and OP wrote the manuscript. All authors have read and approve the final manuscript.
